# Differences in Nanostructure and Hydrophobicity of Cicada (*Cryptotympana atrata*) Forewing Surface with the Distribution of Precipitation

**DOI:** 10.1155/2018/5305847

**Published:** 2018-04-03

**Authors:** Mingxia Sun, Jiajing Zhang, Gregory S. Watson, Jolanta A. Watson, Dong Han, Aiping Liang

**Affiliations:** ^1^Key Laboratory of Systematic and Evolution, Institute of Zoology, Chinese Academy of Sciences, Beichen West Road 1-5, Chaoyang District, Beijing 100101, China; ^2^Zhongguancun Middle School, Kexueyuannan Road 14, Haidian District, Beijing 100080, China; ^3^Faculty of Science, Health, Education and Engineering, University of the Sunshine Coast, Fraser Coast Campus, Hervey Bay, QLD 4655, Australia; ^4^National Center for Nanoscience and Technology, Chinese Academy of Sciences, No. 11, Beiyitiao Zhongguancun, Haidian District, Beijing 100190, China; ^5^College of Life Sciences, University of Chinese Academy of Sciences, Beijing 100049, China

## Abstract

Although the cicada wing has a variety of functions and the nanostructure and surface properties of many species have been extensively investigated, there are no reports investigating diversity of nanostructures and wetting properties within a single species collected at locations with different rainfall conditions. In this study, the hydrophobicity and nanostructure dimensions of the forewing surface of *Cryptotympana atrata* were measured, based on specimens collected from 12 distributions with varying precipitation averages in China and Japan. The relationships among hydrophobicity, nanostructures, and precipitation were analyzed, and the adaption of hydrophobic nanostructures under different wet environments is discussed. The precipitation of locations in the years the samples of *C. atrata* were collected only has an effect on the diameter and spacing of wing surface nanostructure, and the multiple years of precipitation may have an influence on the basic diameter and spacing, as well as the height of protrusions. The rougher the wing surface, the stronger the hydrophobicity which was observed from samples taken where the rainfall conditions of the collection years are high. To our knowledge, this is one special example providing evidence of hydrophobic nanostructures found on a biological surface of a single species which shows adaption for specific wet environments.

## 1. Introduction

Through several billion years of evolution, biological surfaces have diversified into various functional structures. To float on the surface of water, for example, the upper side of the lotus leaf has a hierarchical structure and wax layer [1] which is superhydrophobic and self-cleaning. In contrast, the margins of the leaves have a much larger contact angle (CA) hysteresis with water owing to the existence of flat folds [2]. Geckos on the other hand have evolved micro/nanostructures on their feet promoting high adhesion combining a synergistic effect of the gecko's muscles [3, 4]. The skin of the gecko has also proven to be a multifunctional, hierarchically structured biomaterial capable of various functions, including self-cleaning, low adhesion, superhydrophobicity, antibacterial activity, biocompatibility, and antiwetting [5]. To blend in with natural environments, many biological surfaces also incorporate specific colors (e.g., in the form of spots and patterns [6]). Such colors can result not only from pigmentation but also from patterned structures (e.g., photonic structures [7] and particularly the green color [8]).

There are numerous other examples in nature demonstrating a variety of functions and specific structures. This includes the collection of water from contrasting patterning on the surface of a desert beetle [9], small channels on a cactus [10], high adhesional structure on many mussel species for contacts with substrates on the seashore to prevent tidal and wave removal [11], structures present on the wings of various insects to provide the mechanical and structural support for flight and superhydrophobicity [12–14], and antiadhesional structure of soil fragments on dung beetles [15]. Generally, the varying combinations of the micro/nanostructures and/or the specific chemical components promote or enhance the desired interaction with these naturally occurring contacting surfaces. Thus, examination of these natural templates has practical implications and is often the impetus for many researchers studying such structures and associated mechanistic processes for specific functionality [16].

Many of the features illustrated above are concerned with the interaction of the natural surface with water either to promote or more commonly resist contact. This antiwetting behavior will often depend on the habit and environment of the organism. For example, water repellency of plant leaves is more common in herbaceous species than those in trees and in subtropical regions. Wetlands also appear to have more species with water-repellent leaves when compared to other regions [1]. In addition to plants, flying insects often demonstrate wetting and more often an antiwetting behavior on various body parts (especially wings). Some termite species have finely structured hairs and additional micro/nanostructure-like waxes to aid in flying during rainy periods, while other species have adopted less hydrophobic surfaces for flight when little precipitation is likely to occur [17, 18].

One of the most interesting flying insects studied in recent times is the cicada. Studies have shown that the structure on the wings of these insects is often multifunctional. The wide variety of small-scale structures [19] on these insects has shown a variety of properties/applications including variable wettability, antireflection [20–23], self-cleaning [24], cell growth platforms [25, 26], material strength properties [27], thermoisolative feature [28], and biomimetic fabrication of the nanostructures [29–33]. As well, it has been shown in a number of previous studies that some cicada structures demonstrate control of the interaction with solid bodies (e.g., natural biological contaminants such as pollens as well as hydrocarbons and silica materials) [21, 34]. Such studies, as well as observation of the resistance to environmental decomposition of certain regions of the samples (e.g., wings) during collection [22], have led to the investigation of other biological materials such as various bacteria where the cicada membranes demonstrated an antimicrobial effect [26]. We have found previously that there are large differences in the hydrophobicity of wing surfaces among cicadas [18, 22, 35]. Some species show very weak hydrophobicity with a small water CA, while others exhibit superhydrophobic interactions. The hydrophobicity generally has a positive correlation with the diameter and height of the structure but a negative correlation with the general spacing of features. As well, stronger hydrophobic wing surfaces often contain higher contents of wax [18, 36], while functional groups with higher surface energies can effectively decrease the hydrophobicity of the cicada wing [37]. The hydrophobicity also influences the adhesional force of droplets on the wings with hydrophilic wings showing significantly higher adhesion forces [35, 37].

In this study, we have investigated a widely distributed species of cicada (*Cryptotympana atrata* Fabricius, 1775) collected from various locations demonstrating contrasting wetting (rain) conditions. We have measured differences in the wing nanostructure dimensions and hydrophobicity of forewing surfaces of the same species of cicada found at different locations. The relationship of the wing nanostructure differences and the precipitation (rain) conditions is tested by statistical analyses to explore the hypotheses of evolutionary patterns in relation to nanostructures and wetting environments.

## 2. Materials and Methods

### 2.1. Species and Specimens

The studied species is *C. atrata* (Fabricius, 1775) (Hemiptera: *Cicadidae*) which is widely distributed in China, North Korea, Northern Laos, and Japan. Twelve specimens were collected from Beijing, Hebei, Tianjin, Jiangsu, Zhejiang, Sichuan, Jiangxi, Fujian, and Guangxi in China and Osaka in Japan (Table 1). Among these, three specimens are from Beijing, and the other nine are from other respective locations. All samples were air-dried and preserved at the National Zoological Museum, Institute of Zoology, Chinese Academy of Sciences.

### 2.2. Sample Preparation

**Figure 1:**
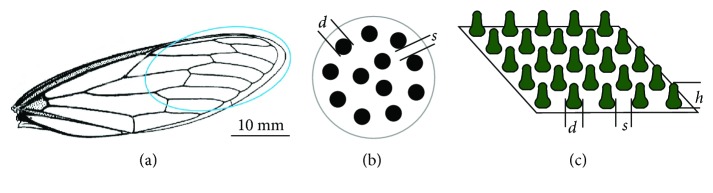
The cicada wing under investigation and the schematic of the wing structures. (a) Image highlighting the test location on the wing (circled). (b, c) Schematic highlighting the dimensional parameters: *d*, *s*, and *h* are the diameter, spacing, and height of protrusions on the cicada wing surface, respectively.

The outermost region of the forewing of *C. atrata* was used in this study as defined in Figure 1(a) (circled region). The forewings of the 12 specimens were washed by flowing deionized water with the end parts excised from the individual samples for further experiments.

### 2.3. Contact Angle Measurement

The hydrophobicity of cicada forewings was measured by placing small water droplets on the surface. The larger the CA, the stronger the hydrophobicity is. If the CA is larger than 90°, the surface is defined as hydrophobic; if it is smaller than 90°, it is defined as hydrophilic. The end parts of the forewings were fixed on a glass slide using double-sided adhesive tape. CA measurements were carried out using a Dataphysics Contact Angle System (OCA 2.0, Germany). The water drop volume was 3 *μ*L. Each sample was measured at 10 sites, with the average value and standard deviation then calculated (Table 1).

### 2.4. Nanostructure Observation and Parameter Calculation

For observing nanostructures of the wing surfaces, the individual samples were fixed on the sample stage using a double-sided adhesive tape. The top surface of the forewing was coated with a thin layer of gold (~10 nm) using ion sputtering (KYKY SBC-12, Beijing, China). Three sites of each sample were selected to observe the basal diameter (*d*), basic spacing (*s*), and height (*h*) of protrusions as illustrated in Figures 1(b) and 1(c) under an environmental scanning electron microscope (Quanta 200 FEG, FEI, Eindhoven, Netherlands). These parameters were measured using Photoshop (version 12.0) software, by utilising the software ruler function and correlating the sizes with the SEM scale bars. Each parameter was measured 20 times to calculate the average value and standard deviation (Table 1).

**Figure 2:**
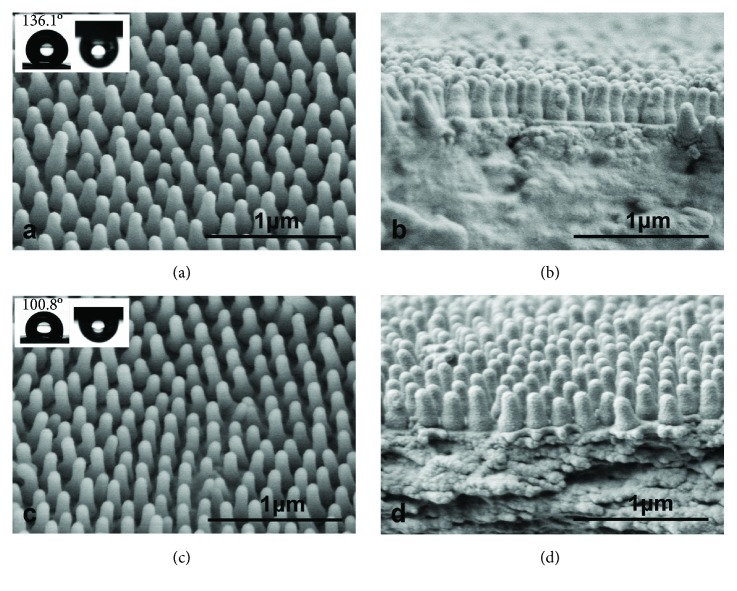
SEM images of protrusions and optical photographs of the contact angles on the cicada *Cryptotympana atrata* wing surfaces. (a) The structure of the sample collected from Zhejiang with a contact angle of 136.1° and large contact angle hysteresis. (b) The cross section of the sample collected from Zhejiang showing the regular arrangement of protrusions. (c) The structure of the sample collected from Fujian with a contact angle of 100.8° and large contact angle hysteresis. (d) The cross section of the sample collected from Fujian showing the quasi-ordered arrangement of protrusions.

For the large CA hysteresis (see inserts in Figures 2(a) and 2(c)), the roughness factor (*γ*) of the wing surfaces, and the actual cicada structure shape as shown diagrammatically in Figures 1(b) and 1(c) calculated here utilize the structural geometry of a cylinder, which describes a very basic, first principle analysis of the Wenzel model [38]:

(1) $$\begin{array}{c} \cos\theta_{w}=\gamma \cos\theta_{0}, \end{array}$$

where *γ* is the roughness factor (the ratio of actual area to geometry projected area of surface), *θ*_0_ is the CA on a smooth surface of the same material, and *θ*_*w*_ is the apparent CA on a rough surface. Thus, the roughness factor (*γ*) of the wing surfaces was calculated using the following, with the results shown in Table 1:

(2) $$\begin{array}{c} \gamma =\frac{{\left(d+s\right)}^{2}+4dh}{{\left(d+s\right)}^{2}}, \end{array}$$

where *d*, *s*, and *h* are the basal diameter, basal spacing, and height of protrusions, respectively.

### 2.5. Precipitation Data and Statistical Methods

The wide distributions of the *C. atrata* cicada species were calibrated by positive latitude and longitude, which reflect the gradient of precipitation from north to south. Rain distribution data for each month of the individual year between 1950 and 2000 and their annual average were obtained from the WorldClim data website (http://www.worldclim.org/current) with a solution of 30′ (~1 × 1 km^2^) and extracted using the PSDS 2.0 software [39]. The annual precipitation of the different locations was then calculated based on the average precipitation of each month from the year 1950 to 2000 (Table S1). The two samples distributed in Japan (collected in 1932) and Beijing North (collected in 2010) are excluded from the collecting data because they were not in the specified range.

Precipitation data for the specific year of *C. atrata* sample collection at the respective locations were downloaded from the China Meteorological Data Sharing Service System website (http://cdc.nmic.cn/home.do), with the exception of Jiangsu as it had no precipitation record in 1951 and Tianjin which did not contain information regarding the collection time (Table S2).

Because many variables do not conform to the normal distribution, Spearman's correlation of the SPSS software (version 18.0) was used for statistical analysis of significant correlations between precipitation, nanostructure, CAs, and the specific year of sample collection (the data are shown in Tables 2 and 3), where *R* is the correlation coefficient and *P* is the significant level, (^∗^ *P* < 0.05 and ^∗∗^ *P* < 0.01), meaning that the two variables have significant correlation. Spearman's analysis shows that precipitation of an individual month or annual precipitation has no correlation with the positive longitude of sample distributions of *C. atrata* (Tables S1 and S2) but significantly correlates to the positive latitude with the exception of precipitation over several months as indicated in Table S1 (*P* < 0.05) and Table S2 (*P* < 0.05). This result is in line with climatic characteristics of China, where precipitation increases when latitude reduces from the north to the south [40]. With the exception of precipitation in July and August (Table S1), January and July to October (Table S2) have no relation to latitude, mainly due to these months showing little differences in precipitation among these various locations.

## 3. Results and Discussion

### 3.1. Relationship between Hydrophobicity and Nanostructure

Observation of the different wing samples (Figure 1(a)) using electron microscopy showed the structure of the *C. atrata* wing surface to be cylindrical in shape, with the approximately spherical apex and significantly enlarged base of the structure (Figures 1(b), 1(c), 2(a), and 2(c)). The diameter (*d*) and height (*h*) of protrusions on the surfaces of cicada wings showed large differences among specimens (diameters (*d*) of 80–130 nm and heights (*h*) of 253–464 nm), but the spacing (*s*) of protrusions showed minimal differences in the range of 67–92 nm. The roughness of wing surfaces, varying from 3.74 to 7.44 nm, indicates large differences among the samples. Consequently, the CAs also varied from 96.1 to 137.9.1° (Table 1), but the CA hysteresis was large, even when the cicada wings were inverted upside down, the water drops still adhered on the surfaces (as in the inserts in Figures 2(a) and 2(c)). Such variations within a species are not usual for biological samples.

**Figure 3:**
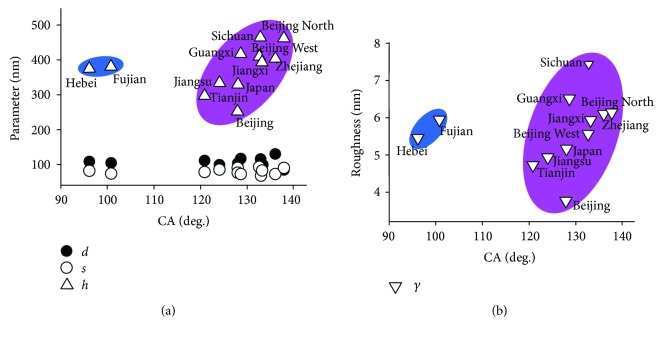
The correlation of contact angle with protrusion parameters on the cicada *Cryptotympana atrata* wing surfaces. (a). Height of protrusion and (b) roughness of the wing surface as a function of contact angle (CA).

Correlation analysis of Spearman's correlation indicated that CA at the tips of the forewings was positively correlated to protrusion height (*P* < 0.05) (Figure 3(a)) and calculated roughness (*P* < 0.05) (Figure 3(b)), especially when the CA is greater than 120°, as indicated by the purple shading in Figure 3. The CA however has no correlation with diameter and spacing between protrusions on the forewing (Figure 3(a)). These results indicate that rougher surfaces have higher hydrophobicity. Samples distributed in Hebei and Fujian (Table 1), as highlighted in blue in Figure 3, have lower CAs of 96.1° and 100.8°, respectively. This is due to inhomogeneous structures resulting from the quasi-ordered arrangement of protrusions on some areas of the wing surfaces (Figure 2(d)) compared to other strictly regular structures (Figure 2(b)).

### 3.2. Relationship of Precipitation with Nanostructure and Life Cycle

**Figure 4:**
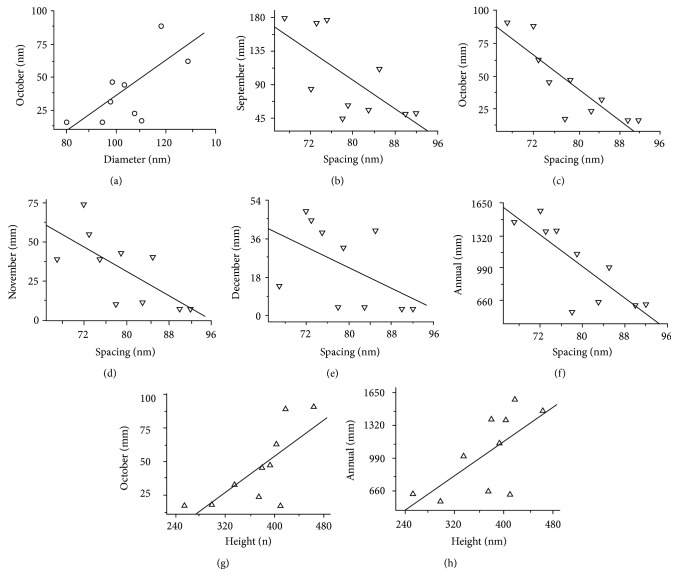
Correlation of the nanostructure with the month and annual average precipitation rates between the years 1950 and 2000, at the various locations the cicada *Cryptotympana atrata* was distributed. (a) The diameter of protrusions shows a positive correlation with the precipitation in October. (b–f) The spacing of protrusions displays a negative relationship with September to December and annual precipitation. (g, h) The height of protrusions shows a positive correlation with the precipitation of October and annual rainfall, respectively. The data were obtained from the WorldClim data.

**Figure 5:**
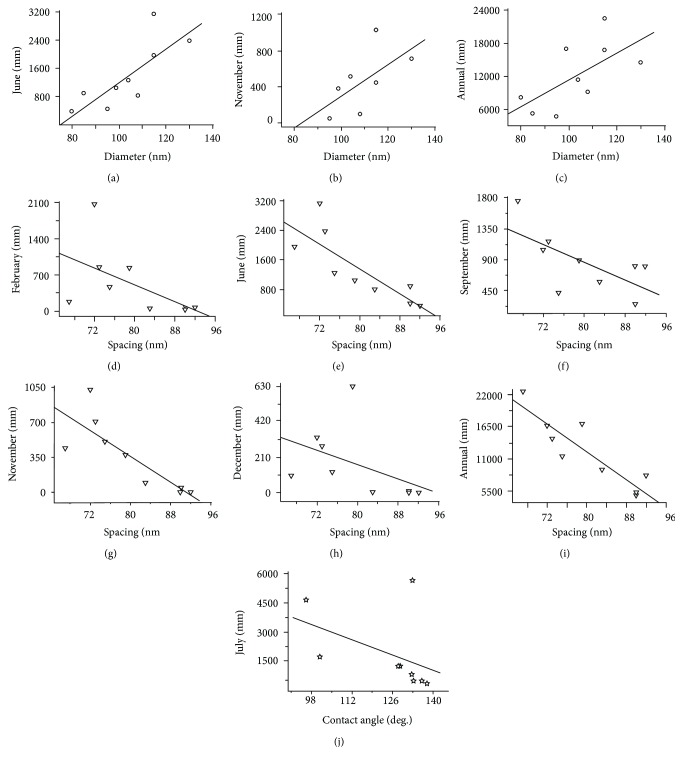
Correlation of protrusion diameter (a–c) with the monthly precipitation of locations and the year the samples of cicada *Cryptotympana atrata* were collected. (a–c) The diameter of nanostructure parameters has a significant correlation with the precipitation in June, November, and annual rainfall. (d–i) The spacing shows significant correlation with the precipitation in February, June, September, November, December, and annual rainfall. (j) The contact angle (CA) shows significant correlation with the precipitation in July. Precipitation data were collected from the China Meteorological data.

Data analysis shows that nanostructural parameters on wing surfaces have significant correlation with precipitations of several months and annual precipitation for the years 1950 to 2000, as per WorldClim data (Table 2). As shown in Figure 4, the diameter of the protrusions shows a positive correlation with the precipitation in October (Figure 4(a)), the spacing displays a negative relationship with three more months from September to December and annual precipitation (Figures 4(b)–4(f)), and the height of protrusions also shows a positive correlation with the precipitation of October (Figure 4(g)) and annual rainfall (Figure 4(h)) (*P* < 0.05), respectively. Meanwhile, using information obtained from the Chinese Meteorological data (Table 3) for the specific month and year of sample collection, the diameter of nanostructure parameters on the wing surfaces does seem to have a significant correlation (*P* < 0.01) with the precipitation in June (Figure 5(a)), November (Figure 5(b)), and annually (Figure 5(c)). The correlation between rain and spacing also seems significant overall months (Figures 5(d)–5(i)), excluding January, March to May, July, August, and October. The height of protrusions however shows no correlation with the precipitation of any month or annual precipitation over the respective collection dates (Table 3). All the three structure parameters including the diameter, spacing, and height of protrusions have no correlation with the specific year of sample collection (the analysis data in the last column of Tables 2 and 3), and this illustrates that the statistical analysis of the precipitation with nanostructure is not affected by the specific year.

The results presented in Tables 2 and 3 show that the precipitation of multiple years between 1950 and 2000 (WorldClim data (Table S1)), and the specific year (China Meteorological data (Table S2)) of the respective locations of the samples of *C. atrata* seems to both have an effect on the diameter and spacing of wing surface nanostructure. Precipitation levels of multiple years appear to have a positive correlation with the height of protrusions and illustrate that the wetting conditions may influence the height of protrusions.

Generally, mature nymphs start to unearth in early June and become adults after ecdysis [41]. The nanoscale protrusions on the wing surface are presumably developed when a nymph becomes an adult cicada (akin to the formation of wing scales when a pupa changes into a butterfly [42]). The precipitation level in June in the specific year of sample collection may have the greatest influence on the formation of the diameter and spacing of protrusions when mature nymphs are undergoing the process of ecdysis. In addition, the cicada pupae usually emerge after living underground for several years; hence, precipitation levels would differ from year to year. As a result, it is unlikely that there would be any direct correlation with a single month's precipitation. Therefore, the diameter, spacing, and height of the wing surface nanostructure could be influenced by precipitation over multiple years, especially in October, when conditions are right for the egg to turn into a nymph, with precipitation levels also having an influence on the development of nanostructure. Similarly, the precipitation a month prior to and two months following October, heavily influencing the development of nanostructures, with the spacing of protrusions (Tables 2 and 3) is influenced by the precipitation during those four or five months. This means that the height of protrusions is possibly a consequence of the accumulated effect of multiple years of rainfall since it has no correlation with the precipitation levels in any month of the specific year the samples were collected. The diameter and spacing are not only the effect of evolution, but is also influenced by the rainfall in that specific year which positively correlates with the trends of precipitation during the year when the samples were collected and multiple years.

### 3.3. Relationship of Precipitation with Hydrophobicity

From Table 2, it can be seen that the precipitation of an individual month and annual rainfall of multiple years shows no correlation with the CA of wing surfaces denoting the hydrophobicity with a great correlation coefficient (*P* = 0.171—0.626). From Table 3, while not the specific year of sample collection (*P* > 0.05), the precipitation during July of the specific year is negatively correlated to the hydrophobic nature of the cicada wing (*P* < 0.01) and has no correlation with the height of nanostructures. The hydrophobicity of cicada wing surfaces is highly correlated with the height of protrusions as shown here and previously [19, 35, 37]. This illustrates the height of protrusions is just a correlative but not a determinant factor.

In wetter conditions (like in the south of China), the bigger the diameter, the smaller the spacing and the greater the height of nanostructure; these three parameters jointly make the wing surface exhibit a higher hydrophobicity than that in drier regions (like in the north of China). Presumably, the samples from wetter areas, with corresponding nanostructures showing a high hydrophobicity, are an evolutionary trait to facilitate a quick removal of water from the wings which would maximize efficiency for movement and flight. In dry conditions, where the rainfall is low and the negative impact of rainfall would be small on flying or movement of insects, the nanostructure of the forewing on the samples had a weak hydrophobicity.

In addition, the hydrophobicity of cicada wing surfaces is also dependent on another aspect besides microstructure, such as chemical components, because surface chemistry is also important in determining hydrophobicity of a solid surface in combination with the microstructure [43, 44].

## 4. Conclusions

In summary, the nanostructure parameters can be seen to significantly affect the hydrophobic properties of cicada wing surfaces. Our study, even though of a small data set, shows significant differences in nanostructure correlated with precipitation at their respective locations. The height of protrusions, roughness of the wing surface, and CA are all related to the precipitation where the species are distributed. The influence of precipitation at the locations during the collection years on the cicada nanostructure diameter and spacing illustrates these two parameters are instantaneously changeable and ambulatory. Conversely, precipitation at locations where the samples were collected over a long period (e.g., 1950–2000) effected the height of nanostructures showing this parameter is constant and evolutionary. Furthermore, the evolution of these nano- and microstructures on a biosurface may be a common phenomenon and worthy of further investigation among populations within diversified taxa. A larger data set of samples collected in the future may corroborate and demonstrate more relationships and subtle differences of these parameters. Given the growing interest in biomimetic material/property development and the knowledge gained in this study, could help in the future design of novel functional biomimetic materials.

## Figures and Tables

**Table 1:** Collection locations, dates, contact angles, and nanostructure parameters of the cicada *Cryptotympana atrata*. The contact angle (CA), basal diameter (*d*), basal spacing (*s*), and height (*h*) values of protrusions are the average values and standard deviations across the forewing of a single specimen. The roughness factor (*γ*) was calculated using (2), which is derived from the Wenzel model. The mark (—) indicates the collecting date of the sample is not recorded.

Species	Locations	Dates	CA	*d*	*s*	*d* + *s*	*h*	*γ*
		D.M.Y	**°**	nm	nm	nm	nm	nm
*C. atrata*	Hebei	11 May 1959	96.1 (5.6)	108 (5)	83 (6)	191	375 (17)	5.44
	Fujian	5 July 1955	100.8 (10.3)	104 (6)	75 (7)	179	380 (27)	5.93
	Tianjin	—	120.9 (11.0)	111 (5)	78 (4)	189	298 (13)	4.70
	Jiangsu	24 July 1951	124.1 (3.6)	98 (4)	85 (9)	183	335 (23)	4.92
	Beijing	18 July 1964	128.0 (1.9)	80 (4)	92 (6)	172	253 (14)	3.74
	Japan	3 August 1932	128.1 (6.4)	103 (5)	78 (6)	181	330 (21)	5.15
	Guangxi	2 July 1985	128.7 (4.5)	115 (3)	72 (6)	187	418 (30)	6.50
	Beijing West	11 August 1951	132.7 (4.0)	95 (5)	90 (8)	185	410 (49)	5.55
	Sichuan	31 July 1989	132.9 (3.2)	115 (7)	67 (4)	182	464 (30)	7.44
	Jiangxi	14 July 1957	133.3 (7.5)	99 (7)	79 (7)	178	393 (20)	5.91
	Zhejiang	3 August 1961	136.1 (4.3)	130 (5)	73 (3)	203	403 (24)	6.09
	Beijing North	25 August 2010	137.9 (1.9)	85 (5)	90 (8)	175	462 (34)	6.13

**Table 2:** A statistical analysis of structure parameters and contact angle (CA) of the *Cryptotympana atrata* cicada with the average monthly and annual precipitation rates from the year 1950 to 2000 of the various cicada distributions. Structure parameters include basal diameter (*d*), basal spacing (*s*), and height (*h*) of protrusions on the wing surfaces. The year refers to the specific year of sample collection. *R*: correlation coefficient; *P*: significant level; ^∗^ *P* < 0.05 and ^∗∗^ *P* < 0.01: the significant correlation.

Parameter		Average precipitations of each month from the year 1950 to 2000												Annual rainfall	Year
		Jan.	Feb.	Mar.	Apr.	May.	Jun.	Jul.	Aug.	Sept.	Oct.	Nov.	Dec.		
*d*	*R*	0.437	0.355	0.327	0.407	0.456	0.328	−0.171	−0.146	0.456	0.750^∗^	0.602	0.587	0.559	0.396
	*P*	0.207	0.315	0.356	0.243	0.185	0.354	0.637	0.687	0.185	0.012	0.065	0.074	0.093	0.257
*s*	*R*	−0.522	−0.500	−0.524	−0.600	−0.600	−0.503	0.207	0.036	−0.648^∗^	−0.888^∗∗^	−0.640^∗^	−0.634^∗^	−0.770^∗∗^	−0.462
	*P*	0.122	0.141	0.120	0.067	0.067	0.138	0.567	0.920	0.043	0.001	0.046	0.049	0.009	0.179
*h*	*R*	0.374	0.427	0.439	0.600	0.539	0.430	−0.073	0.146	0.491	0.693^∗^	0.457	0.366	0.661^∗^	0.474
	*P*	0.287	0.219	0.204	0.067	0.108	0.214	0.841	0.688	0.150	0.026	0.184	0.298	0.038	0.166
CA	*R*	0.460	0.470	0.201	0.370	0.442	0.297	−0.310	−0.176	0.248	0.456	0.409	0.238	0.309	0.377
	*P*	0.181	0.171	0.577	0.293	0.200	0.405	0.383	0.626	0.489	0.185	0.241	0.508	0.385	0.283

**Table 3:** A statistical analysis of the structure parameters and contact angle (CA) with the precipitation rates of the month and year the various samples of cicada *Cryptotympana atrata* were collected. Structure parameters include basal diameter (*d*), basal spacing (*s*), and height (*h*) of protrusions on the wing surfaces. The year refers to the specific year of sample collection. *R*: correlation coefficient; *P*: significant level; ^∗^ *P* < 0.05  and ^∗∗^ *P* < 0.01: the significant correlation.

Parameter		The precipitations of each month in the years of different samples collected												Annual rainfall	Year
		Jan.	Feb.	Mar.	Apr.	May.	Jun.	Jul.	Aug.	Sept.	Oct.	Nov.	Dec.		
*d*	*R*	0.510	0.653	0.569	0.259	0.293	0.837^∗∗^	0.410	0.067	0.603	0.619	0.862^∗∗^	0.605	0.669^∗^	0.092
	*P*	0.160	0.057	0.110	0.500	0.444	0.005	0.273	0.864	0.086	0.075	0.003	0.084	0.049	0.814
*s*	*R*	−0.661	−0.711^∗^	−0.661	−0.510	−0.485	−0.921^∗∗^	−0.444	−0.100	−0.686^∗^	−0.594	−0.870^∗∗^	−0.693^∗^	−0.845^∗∗^	−0.201
	*P*	0.053	0.032	0.053	0.160	0.185	0.000	0.232	0.797	0.041	0.092	0.002	0.038	0.004	0.604
*h*	*R*	0.383	0.133	0.150	−0.017	0.117	0.500	−0.083	0.000	0.517	0.467	0.150	0.142	0.250	0.517
	*P*	0.308	0.732	0.700	0.966	0.765	0.170	0.831	1.000	0.154	0.205	0.700	0.715	0.516	0.154
CA	*R*	0.567	0.100	0.367	−0.033	0.133	0.267	−0.717^∗^	0.250	0.533	0.633	−0.100	0.151	0.117	0.400
	*P*	0.112	0.798	0.332	0.932	0.732	0.488	0.030	0.516	0.139	0.067	0.798	0.699	0.765	0.286

## Supplementary Materials

Supplementary Materials Supplementary materials associated with this article can be found in the attached file. Table S1: a statistical analysis of the positive latitude and longitude with the average monthly and annual precipitation rates from 1950 to 2000 of the various *Cryptotympana atrata* cicada distributions. The sample distributions were calibrated by positive latitude and longitude. The data were downloaded from the WorldClim (WC) data website (http://www.worldclim.org/current) at the 30 arcseconds (~1 × 1 km^2^) resolution. *R*: correlation coefficient; *P*: significant level; ^∗^ *P* < 0.05 and ^∗∗^ *P* < 0.01: the significant correlation. Table S2: a statistical analysis of the positive latitude and longitude with the precipitation rates of the month and year of the various samples of *Cryptotympana atrata* cicada was collected. The precipitation rates for the particular collection year were downloaded from the China Meteorological (CM) Data Sharing Service System website (http://cdc.nmic.cn/home.do). *R*: correlation coefficient; *P*: significant level; ^∗^ *P* < 0.05 and ^∗∗^ *P* < 0.01: the significant correlation.
