# Supplementary material for: Differences in Nanostructure and Hydrophobicity of Cicada (Cryptotympana atrata) Forewing Surface with the Distribution of Precipitation
Source: Appl Bionics Biomech. 2018 Apr 3;2018:5305847. doi: 10.1155/2018/5305847 (PMC5903195; doi:10.1155/2018/5305847)
Supplement: Supplementary Materials — associated with this article can be found in the attached file. Table S1: a statistical analysis of the positive latitude and longitude with the average monthly and annual precipitation rates from 1950 to 2000 of the various Cryptotympana atrata cicada distributions. The sample distributions were calibrated by positive latitude and longitude. The data were downloaded from the WorldClim (WC) data website (http://www.worldclim.org/current) at the 30 arcseconds (~1 × 1 km2) resolution. R: correlation coefficient; P: significant level; ∗ P < 0.05 and ∗∗ P < 0.01: the significant correlation. Table S2: a statistical analysis of the positive latitude and longitude with the precipitation rates of the month and year of the various samples of Cryptotympana atrata cicada was collected. The precipitation rates for the particular collection year were downloaded from the China Meteorological (CM) Data Sharing Service System website (http://cdc.nmic.cn/home.do). R: correlation coefficient; P: significant level; ∗ P < 0.05 and ∗∗ P < 0.01: the significant correlation. [file 5305847.f1.doc]

**Supplementary Materials**

**Table S1. A statistical analysis of the positive latitude and longitude with the average monthly and annual precipitation rates from 1950 – 2000 of the various *Cryptotympana atrata* cicada distributions. The sample distributions were calibrated by positive latitude and longitude. The data were downloaded from the WorldClim (WC) data website (**[***http://www.worldclim.org/***](http://www.worldclim.org/) ***current*) at the 30 arc-seconds (~1×1 km^2^) resolution. R, correlation coefficient and P, significant level; * (P < 0.05) and ** (P < 0.01), the significant correlation.**

| **Locations** | **Positive latitude and longitude** | **Average precipitations of each month from the year 1950 to 2000（mm）** | | | | | | | | | | | | **Annual rainfall**  **（mm）** |
| --- | --- | --- | --- | --- | --- | --- | --- | --- | --- | --- | --- | --- | --- | --- |
|  |  | **Jan.** | **Feb.** | **Mar.** | **Apr.** | **May.** | **Jun.** | **Jul.** | **Aug.** | **Sept.** | **Oct.** | **Nov.** | **Dec.** |  |
| Beijing | 39°58'45.09" N 116°18'57.45" E | 4 | 5 | 9 | 19 | 33 | 74 | 204 | 195 | 51 | 16 | 7 | 3 | 620 |
| Beijing West | 39°59'53.03" N 116°20'26.93" E | 4 | 5 | 9 | 20 | 32 | 73 | 201 | 195 | 50 | 16 | **7** | 3 | 615 |
| Hebei | 39°42'46.14" N 119°9'45.73" E | 3 | 4 | 12 | 23 | 48 | 76 | 204 | 180 | 56 | 23 | 11 | 4 | 644 |
| Tianjin | 39°7'15.15" N 117°12'54.11" E | 4 | 4 | 8 | 17 | 31 | 68 | 183 | 154 | 44 | 17 | 10 | 4 | 544 |
| Jiangsu | 31°33'58.13"N  120°18'10.90"23E | 47 | 62 | 59 | 74 | 98 | 152 | 163 | 126 | 111 | 32 | 40 | 40 | 1004 |
| Zhejiang | 30°16'24.59" N  120° 9'18.90" E | 63 | 88 | 108 | 122 | 168 | 203 | 136 | 147 | 173 | 63 | 55 | 45 | 1371 |
| Sichuan | 29°34'46.58" N 103°23'2.97" E | 15 | 23 | 39 | 80 | 128 | 148 | 343 | 364 | 180 | 91 | 39 | 14 | 1464 |
| Jiangxi | 28°31'59.34" N 115°56'55.72" E | 44 | 64 | 109 | 141 | 182 | 213 | 108 | 95 | 62 | 47 | 43 | 32 | 1140 |
| Fujian | 26° 1'34.37" N 119°22'55.65" E | 46 | 87 | 124 | 137 | 158 | 204 | 142 | 181 | 177 | 45 | 39 | 39 | 1379 |
| Guangxi | 25°51'25.07" N 110°29'15.02"E | 58 | 83 | 124 | 177 | 281 | 252 | 158 | 152 | 84 | 89 | 74 | 49 | 1581 |
| Longitude | R | 0.202 | 0.152 | 0.012 | -0.188 | -0.188 | -0.091 | -0.304 | -0.365 | 0.055 | -0.292 | 0.018 | 0.201 | -0.273 |
|  | P | 0.575 | 0.674 | 0.973 | 0.603 | 0.603 | 0.803 | 0.393 | 0.300 | 0.881 | 0.413 | 0.960 | 0.577 | 0.446 |
| Latitude | R | -0.706^*^ | -0.738^*^ | -0.884^**^ | -0.891^**^ | -0.867^**^ | -0.879^**^ | 0.571 | 0.365 | -0.673^*^ | -0.839^**^ | -0.817^**^ | -0.811^**^ | -0.867^**^ |
|  | P | 0.023 | 0.015 | 0.001 | 0.001 | 0.001 | 0.001 | 0.084 | 0.300 | 0.033 | 0.002 | 0.004 | 0.004 | 0.001 |

**Table S2** A statistical analysis of the positive latitude and longitude with the precipitation rates of the month and year the various samples of cicada *Cryptotympana atrata* were collected. The precipitation rates for the particular collection year were downloaded from the China Meteorological (CM) Data Sharing Service System website *(*[*http://cdc.nmic.cn/home.do*](http://cdc.nmic.cn/home.do)). R, correlation coefficient; P, significant level; * (P < 0.05) and ** (P < 0.01), the significant correlation.

| **Locations** | **Years** | **The precipitations of each month in the years of different samples collected (mm)** | | | | | | | | | | | | **Annual rainfall**  **（mm）** |
| --- | --- | --- | --- | --- | --- | --- | --- | --- | --- | --- | --- | --- | --- | --- |
|  |  | **Jan.** | **Feb.** | **Mar.** | **Apr.** | **May.** | **Jun.** | **Jul.** | **Aug.** | **Sept.** | **Oct.** | **Nov.** | **Dec.** |  |
| Beijing North | 2010 | 104 | 26 | 222 | 175 | 295 | 887 | 340 | 1778 | 808 | 590 | 0 | 0 | 5225 |
| Beijing | 1964 | 75 | 59 | 106 | 1416 | 132 | 366 | 1249 | 3573 | 806 | 388 | 1 | 0 | 8171 |
| Beijing West | 1951 | 82 | 102 | 14 | 9 | 1458 | 431 | 822 | 1231 | 258 | 355 | 45 | 8 | 4815 |
| Hebei | 1959 | 0 | 52 | 133 | 201 | 85 | 813 | 4637 | 2004 | 580 | 576 | 94 | 3 | 9178 |
| Zhejiang | 1961 | 617 | 855 | 1090 | 758 | 2056 | 2377 | 482 | 2633 | 1166 | 1465 | 711 | 277 | 14487 |
| Sichuang | 1989 | 251 | 187 | 581 | 1780 | 1101 | 1961 | 5648 | 7477 | 1763 | 1253 | 444 | 101 | 22547 |
| Jiangxi | 1957 | 1215 | 832 | 1485 | 3351 | 3008 | 1045 | 480 | 2658 | 895 | 1028 | 375 | 631 | 17003 |
| Fujian | 1955 | 185 | 472 | 805 | 922 | 3791 | 1251 | 1735 | 1223 | 421 | 1 | 509 | 122 | 11437 |
| Guangxi | 1985 | 274 | 2063 | 1064 | 2276 | 2231 | 3126 | 1250 | 1489 | 1048 | 596 | 1029 | 327 | 16773 |
| Longitude | R | -0.250 | -0.067 | -0.100 | -0.567 | 0.033 | -0.183 | -0.100 | -0.400 | -0.483 | -0.333 | 0.033 | -0.126 | -0.450 |
|  | P | 0.516 | 0.865 | 0.798 | 0.112 | 0.932 | 0.637 | 0.798 | 0.286 | 0.187 | 0.381 | 0.932 | 0.748 | 0.224 |
| Latitude | R | -0.617 | -0.817** | -0.717* | -0.767* | -0.717* | -0.733* | -0.400 | 0.083 | -0.350 | -0.217 | -0.867** | -0.820** | -0.783** |
|  | P | 0.077 | 0.007 | 0.030 | 0.016 | 0.030 | 0.025 | 0.286 | 0.831 | 0.356 | 0.576 | 0.002 | 0.007 | 0.013 |
